# Primary health care professionals’ opinions regarding continuing medical education: A cross sectional study

**DOI:** 10.1097/MD.0000000000040865

**Published:** 2024-12-13

**Authors:** Hassan Mohammed Hassan Alshehri, Ali Mohammed Hassan Alshehri, Amer Mohammed Hassan Alshehri, Salem Mohammed Hassan Alshehri, Yousef H. Al Zahib, Ayedh A.A. Alahmari, Saleh Ahmed Alshaikhi, Hassan Mohammed Algarni, Fahad Saeed Nasser Alasmari

**Affiliations:** a Department of Family Medicine, Ministry of Health, Riyadh, Saudi Arabia; b Department of Family Medicine, Ministry of Health, Abha, Saudi Arabia; c Department of Family Medicine, Ministry of Health, Muhayil Aseer, Saudi Arabia; d Department of Dentistry, Ministry of Health, Bariq, Saudi Arabia; e Department of Family Medicine, Ministry of Health, Khamis Mushait, Saudi Arabia; f Department of Family Medicine, Ministry of Health, Jeddah, Saudi Arabia; g Department of Dentistry, Ministry of Health, Riyadh, Saudi Arabia.

**Keywords:** continuing medical education, health personnel, knowledge, primary health care, professional competence

## Abstract

Continuing medical education (CME) is the foremost among the learning strategies that enhance knowledge and skills within the medical profession. This study aimed to evaluate primary healthcare professionals’ CME in Abha City. A total of 267 primary health care (PHC) professionals. The researcher designed a data collection sheet that comprised personal characteristics and aspects of CME. About two-thirds of participants (65.5%) attended CME courses during the last year. The main criteria for attending CME courses were their topics (43.4%), convenience of time (41.2%), and interests (31.5%). About 16.1% of participants found that previously attended CME courses were not useful because of being a waste of time (12.7%), not obtaining any benefit (7.9%), and wasting of money (3.7%). On the other hand, 83.9% found that previously attended CME courses were useful because of their improved knowledge (68.9%) and skills (47.6%), while 30.7% stated that attending CME courses was to fulfill the required CME hours. Lectures were the most preferred educational method(48.3%). Hospital consultants were mostly preferred speakers (53.6%), followed by PHC physicians (34.1%) and university staff (25.8%). The main participants’ source of information (95.5%), followed by textbooks (25.1%) and their colleagues at PHC (12.4%). About 31.5% of participants had no or low improvement in their knowledge, 38.6% underwent intermediate improvement and 30% had high improvement. Regarding improved skills, 43.4% had no or low improvement, 45.3% had intermediate improvement and 11.2% had high improvement. The main barriers against attending CME courses were the lack of time (44.9%) and the high workload (41.2%). CME attendance differed significantly according to participants’ position (*P* = .023), with physicians being the most frequently attending (72.9%) and pharmacists being the least frequently attending (36.4%). Participants’ knowledge improvement due to CME attendance differed significantly according to their position, with the highest improvement among lab technicians. Participants’ skills improvement was highest among those aged > 40 years, among males, non-Saudis, those with Doctorate degrees, physicians, and those with > 10 years’ experience. PHC professionals find CME courses valuable for knowledge enhancement, with lectures, videos, and presentations preferred. Information sources include internet, textbooks, and colleagues, but time constraints and workloads hinder attendance.

## 1. Introduction

A competent physician has a professional responsibility to continue to learn throughout his careers, to maintain and improve his knowledge and skills in order to provide safe and effective health care for his patients.^[1,2]^ Without a program of active learning, no physician will be able to remain competent for more than a few years after graduation.^[3]^ Grant^[4]^ noted that physicians who fail to acquire new knowledge and skills might, after 10 years, be only 25% as efficient as they were at the time of graduation.

Continuing medical education (CME) is the foremost among the learning strategies that enhance knowledge and skills within the medical profession.^[5]^ Davis^[6]^ defined CME as any way by which doctors learn after the formal completion of their training. The primary purpose of CME is to keep professionals up-to-date with the latest knowledge in their profession and to enable competent practice for the benefit of patient care.^[3,7]^ CME involves educational activities to maintain, develop, or increase a physician’s knowledge, skills, performance, and relationships, including conferences, workshops, symposia, courses, and departmental targeted meetings, while also incorporating personal efforts for professional development.^[8]^

For all health professionals, regardless of discipline, specialty or type, maintaining professional competence is a core responsibility.^[2]^ Although formal CME may be a recent phenomenon, the concept of CME is not new. Health professionals have been involved in some form of CME since the early 20th century.^[9]^

The Kingdom of Saudi Arabia is not different from other parts of the world, in which its human capital development in health sciences is facing several challenges and obstacles that need to be studied from different perspectives. Saudi Arabia introduced an accreditation system in 1995, counting CME activities for licensing and relicensing. The Saudi Commission for Health Profession (SCHS) regulates health professionals and related medical education programs, with a steady increase in accredited CME programs since 2002.^[10]^ The Saudi Commission for Health Specialties (SCFHS) approves and accredits all CME in Saudi Arabia, requiring healthcare practitioners to acquire specific CME hours for relicensing to practice. There are several challenges that face Saudi CME. These are coherence between educational activities and work experience, cooperation and co-ordination among multiple providers, and resources, assessment of needs, effectiveness and quality control.^[9]^

CME is essential for the professional development of healthcare providers, especially as it has become a global mandate to keep pace with the rapidly evolving healthcare landscape. However, in Saudi Arabian hospitals, CME faces challenges that hinder these programs from fully addressing the needs of healthcare professionals and adapting to the complexities of modern health care delivery systems.^[11]^ To the best of the researcher’s knowledge, no recent studies have been conducted in the past decade in the Aseer Region to assess the needs and practices related to CME among PHC professionals. This study, therefore, aims to evaluate the CME practices and needs of PHC professionals in Abha City, providing insights into how CME can better support this workforce in their ongoing professional development.

## 2. Subjects and methods

### 2.1. Study design

Cross sectional study.

### 2.2. Study area

Primary health care (PHC) centers in Abha City, Kingdom of Saudi Arabia.

### 2.3. Study population and sampling

The study population included all healthcare professionals at 47 primary healthcare centers in Abha City.^[12]^ A multistage stratified random sampling method was employed to select participants for the study. In the first stage, PHC centers in Abha City were stratified based on geographic location (e.g., central, northern, southern, etc) to ensure that different regions within the city were represented. A random sample of centers from each geographic stratum was then selected. In the second stage, healthcare professionals within each selected center were further stratified by their professional roles (e.g., physicians, pharmacists, nurses, lab technicians, and dentists) to reflect the diversity of professional categories. A random sample of individuals was then drawn from each professional group within each selected center. The following table shows the total number of healthcare professionals, the number of included participants and the corresponding response rates according to participants’ positions: Among the 295 total participants, 267 responded. Physicians had the highest response rate, with 48 of 52 (92.3%) participating. Nurses, who represented the largest group, also demonstrated a high response rate of 91.8%, with 167 of 182 responding. Pharmacists had a response rate of 88.0%, with 22 of 25 participating, while dentists had a similar response rate of 87.0%, with 20 of 23 responding. Laboratory technicians had the lowest response rate at 76.9%, with 10 of 13 included.

Exclusion Criteria: The study excluded healthcare professionals who were on extended leave (e.g., maternity leave, medical leave, or long-term vacation) during the data collection period, as well as the temporary or part-time staff who were not consistently available in the PHC centers. Additionally, administrative staff without direct patient care responsibilities were excluded, as the study focused specifically on the CME needs of clinical healthcare providers.

Data collection tool: See Annex, Supplemental Digital Content, http://links.lww.com/MD/O138.

After obtaining all the necessary official approvals, the study questionnaires were distributed to all healthcare professionals in all PHC centers in Abha City. The researcher designed a data collection sheet that comprises the following 2 parts:

1- Personal characteristics: age, gender, nationality, marital status, position, qualification and years of experience in PHC.

2- Aspects of continuing education: based on review of relevant literature, the researcher constructed questions about continuing education of health care providers (See Annex). It comprised items, i.e., attending CME activities, important factors for selecting CME activity, preferred educational delivery methods, preferred speakers, main resources for CME, barriers against selecting a CME activity, and perceived impact of attended CME activities on development of participants’ knowledge and skills [Supplemental Digital Content (study questionnaire), http://links.lww.com/MD/O138].

## 3. Pilot study

The researcher conducted a pilot study on 20 PHC professionals. The pilot study helped in finalization of the study questionnaire and assessment of the reliability of its statements. Data collected within the pilot study were not be included in the main study.

## 4. Data entry and statistical analysis

Data were entered to a personal computer and were analyzed using the Statistical Package for the Social sciences (SPSS version 21). Statistical tests of significance, (e.g., Chi-square) were applied. *P*-values <.05 were considered as statistically significant.

## 5. Administrative and ethical considerations

All necessary official approvals to conduct this study were obtained. The individual consent from each patient to participate in the study was a prerequisite for data collection. All collected data were kept confidential and were not accessed except for the purpose of research.

## 6. Budget

This study was completely self-funded by the researcher.

## 7. Potential bias

The methodology of this study can introduce potential biases such as selection bias, nonresponse bias, cluster bias, sampling frame bias, and geographic bias. These can affect the generalizability of findings and may result in an incomplete understanding of specific groups or practices.

## 8. Results

Table 1 shows that 49.8% were aged 30 to 40 years 55.1% were females, 88.8% were Saudi, 65.2% were married, 86.9% had a bachelor’s degree, 33.7% had <5 years’ experience in primary care, and 57.3% had 5 to 10 years’ experience in primary care. 65.5% of participants attended CME courses during the last year. CME was attended once by 11.6%, 2 to 3 times by 40.8% and more than 3 times by 13.1% of participants.

**Table 1:** Personal characteristics of study sample.

Characteristics	No.	%
Age groups		
<30 yr	98	36.7
30 to 40 yr	133	49.8
>40 yr	36	13.5
Gender		
Male	120	44.9
Female	147	55.1
Nationality		
Saudi	237	88.8
Non-Saudi	30	11.2
Marital status		
Single	93	34.8
Married	174	65.2
Qualifications		
Bachelor	232	86.9
Diploma	13	4.9
MSc	14	5.2
Doctorate	8	3.0
Years of experience		
<5 yr	90	33.7
5 to 10 yr	153	57.3
Attending CME		
No	92	34.5
Yes	175	65.5
1	31	11.6
2 to 3	109	40.8
>3	35	13.1

Abbreviation: CME = continuing medical education.

**Figure 1.**
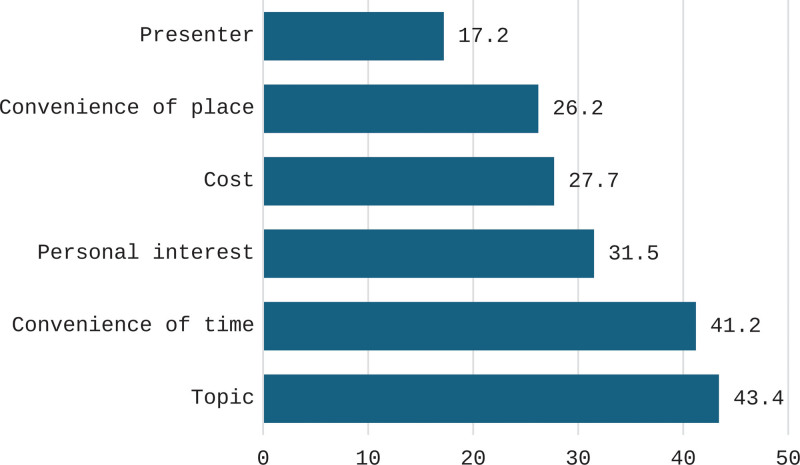
The main criteria for attending CME courses. CME = continuing medical education.

Figure 1 shows that the main criteria for attending CME courses were their topics (43.4%), convenience of time (41.2%) and participants’ personal interests (31.5%).

Table 2 shows that 16.1% of participants found that previously attended CME courses were not useful, mainly because of being waste of time (12.7%), not obtaining any actual benefit (7.9%) and waste of money (3.7%). On the other hand, 83.9% of participants found that previously attended CME courses were useful, mainly because of their improved knowledge (68.9%) and skills (47.6%), while 30.7% stated that attending CME courses was to fulfill the required CME hours.

**Table 2:** Participants’ opinions regarding evaluation of previously attended CME.

Opinions	No.	%
Not useful, why:	43	16.1
Waste of time	34	12.7
No actual benefit	21	7.9
Waste of money	10	3.7
Useful, why:	224	83.9
Improves knowledge	184	68.9
Improves skills	127	47.6
To fulfill required CME hours	82	30.7

Abbreviation: CME = continuing medical education.

**Figure 2.**
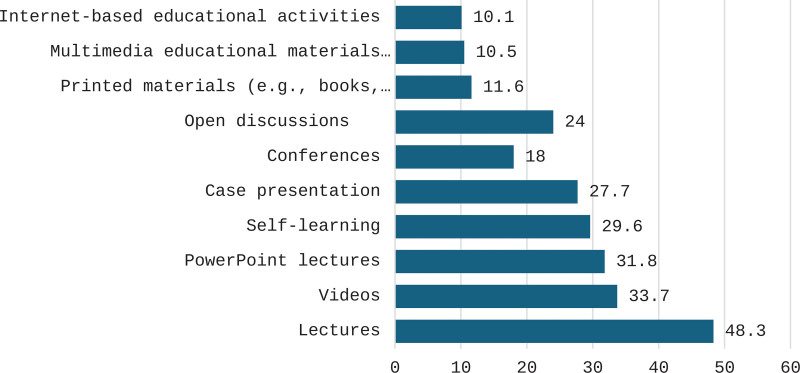
The most preferred educational methods by participants.

Figure 2 shows lectures were the most preferred educational methods by participants (48.3%), followed by videos (33.7%) and PowerPoint lectures (31.8%).

Table 3 shows hospital consultants were mostly preferred speakers by participants (53.6%), followed PHC physicians (34.1%) and university staff (25.8%), while technical supervisors were the least preferred (15%). The main participants’ source of information (95.5%), followed by textbooks (25.1%) and their colleagues at PHC (12.4%). The least source for information was medical journals (5.6%). Around 31.5% of participants had no or low improvement in their knowledge, 38.6% underwent intermediate improvement and 30% had high improvement. Regarding improved skills, 43.4% had no or low improvement, 45.3% had intermediate improvement and 11.2% had high improvement. The main barriers against attending CME courses were the lack of time (44.9%) and the high workload (41.2%).

**Table 3:** Preferences for continuing medical education (CME) speakers, information sources, knowledge and skill improvements, and barriers to attendance.

		**No.**	**%**
Preferred Speakers	Hospital consultants	143	53.6
	PHC physicians	91	34.1
	University staff	69	25.8
	Technical supervisors	40	15
Main sources of information	Internet	255	95.5
	Textbooks	67	25.1
	Colleagues at PHC	33	12.4
	Medical journals	15	5.6
Improvement in knowledge	None/low	84	31.5
	Intermediate	103	38.6
	High	80	30
Improvement in skills	None/low	116	43.4
	Intermediate	121	45.3
	High	30	11.2
Barriers against attending CME courses	Lack of time	120	44.9
	High workload	110	41.2
	Lack of money	62	23.2
	Family and social obligations	50	18.7

Abbreviations: CME = continuing medical education, PHC = primary health care.

Table 4 shows that CME attendance differed significantly according to participants’ position (*P* = .023), with physicians being the most frequently attending (72.9%) and pharmacists being the least frequently attending (36.4%). However, CME attendance did not differ significantly according to other personal characteristics.

**Table 4:** Participants’ CME attendance during the last year according to their characteristics.

Characteristics	Attended CME		Did not attend CME		*P*-value
	No.	%	No.	%	
Age groups					
<30 yr	98	67.1	48	32.9	.303
30 to 40 yr	65	61.3	41	38.7	
>40 ys	12	80.0	3	20.0	
Gender					
Male	42	60.0	28	40.0	.256
Female	133	67.5	64	32.5	
Nationality					
Saudi	153	64.6	84	35.4	.341
Non-Saudi	22	73.3	8	26.7	
Marital status					
Single	62	66.7	31	33.3	.778
Married	113	64.9	61	35.1	
Qualifications					
Bachelor	149	64.2	83	35.8	.375
Diploma	8	61.5	5	38.5	
MSc	12	85.7	2	14.3	
Doctorate	6	75.0	2	25.0	
Position					
Physician	35	72.9	13	27.1	.023
Pharmacist	8	36.4	14	63.6	
Dentist	11	55.0	9	45.0	
Lab technician	6	60.0	4	40.0	
Nurse	115	68.9	52	31.1	
Years of experience					
<5 yr	51	56.7	39	43.3	.081
5 to 10 yr	106	69.3	47	30.7	
>10 yr	18	75.0	6	25.0	

Abbreviation: CME = continuing medical education.

Table 5 shows that participants’ skills improvement due to CME attendance differed significantly according to their age (*P* = .009), with highest improvement among those aged > 40 years. It also differed significantly according to their gender (*P* < .001), with higher improvement among males. It differed according to their nationality (*P* = .004), with higher improvement among non-Saudis. It differed according to participants’ qualifications (*P* = .03), with highest improvement among those with Doctorate degrees. It differed significantly according to their position (*P* < .001), with highest improvement among physicians. Finally, it differed significantly according to their years of experience in primary care (*P* < .001), with highest improvement among those with more than 10 years’ experience.

**Table 5:** Participants’ skills improvement after CME attendance according to their personal characteristics.

Characteristics	None/low		Intermediate		High		*P*
	No.	%	No.	%	No.	%	value
Age groups							
<30 yr	45	30.8	60	41.1	41	28.1	.009
30 to 40 yr	39	36.8	38	35.8	29	27.4	
>40 yr	0	0.0	5	33.3	10	66.7	
Gender							
Male	10	14.3	24	34.3	36	51.4	<.001
Female	74	37.6	79	40.1	44	22.3	
Nationality							
Saudi	81	34.2	92	38.8	64	27.0	.004
Non-Saudi	3	10.0	11	36.7	16	53.3	
Marital status							
Single	40	43.0	34	36.6	19	20.4	.005
Married	44	25.3	69	39.7	61	35.1	
Qualifications							
Bachelor	73	31.5	92	39.7	67	28.9	.030
Diploma	4	30.8	7	53.8	2	15.4	
MSc	7	50.0	2	14.3	5	35.7	
Doctorate	0	0.0	2	25.0	6	75.0	
Position							
Physician	0	0.0	9	18.8	39	81.3	<.001
Pharmacist	0	0.0	12	54.5	10	45.5	
Dentist	0	0.0	9	45.0	11	55.0	
Lab technician	2	20.0	6	60.0	2	20.0	
Nurse	82	49.1	67	40.1	18	10.8	
Years of experience							
<5 yr	25	27.8	25	27.8	40	44.4	<.001
5 to 10 yr	53	34.6	72	47.1	28	18.3	
>10 yr	6	25.0	6	25.0	12	50.0	

Abbreviation: CME = continuing medical education.

## 9. Discussion

Physicians who have completed training and are deemed qualified to practice are expected to maintain competency and consequently spend a considerable amount of time engaged in CME.^[13]^ A competent health care professional should always have professional responsibility to continue to learn throughout his/her career, to maintain and improve their knowledge and skills in order to provide safe and effective health care for patients.^[14]^ Therefore, the current study aimed to explore practices and needs of PHC professionals in Abha City regarding CME.

The findings of this study showed that 65.5% of participants attended CME courses during the last year. CME was attended once by 11.6%, 2 to 3 times by 40.8% and more than 3 times by 13.1% of participants. The finding of the present study is in accordance with those of Shahabudin and Edariah,^[15]^ in Malaysia, who reported that 78% of physicians indicated that they were participating in CME. In Alahsa, Saudi Arabia, Alhejji et al^[16]^ reported that 76.9% of primary care physicians practised CME.

The current study showed that the main criteria for attending CME courses were their topics, convenience of time and participants’ interests. Similarly, Bower^[13]^ stressed that the topic and time of CME are important preferred criteria that would encourage to attend or to miss attendance. Moreover, Stueland-Adamski^[17]^ indicated that irrelevance of topics or poor quality of courses were their reasons for nonattendance, while nearby location was important to 22.3% of respondents and convenience was important to 18.4%.

The present study revealed that 16.1% of participants found that attending CME courses was not useful, mainly because of is a waste of time, not obtaining any actual benefit and a waste of money. On the other hand, 83.9% of participants found that previously attended CME courses were useful, mainly because of their improved knowledge and skills, while 30.7% stated that attending CME courses was to fulfill the required CME hours. Al-Mosilhi and Kurashi^[18]^ stressed the importance of CME for primary care physicians. They stated that PHC is essential for the improvement of community health and CME helps all physicians to keep up-to-date. The explosion of scientific information, rapidly advancing medical concepts and the rapid changes in technologies, underscore the inevitability of useful CME programs for PHC physicians. However, Alghamdi^[11]^ reported that CME for physicians in Saudi Arabia needs to be organized to avoid waste of time and money and to ensure optimum utilization of available resources.

The present study showed lectures were the most preferred educational methods by participants, followed by videos and PowerPoint presentations. In Aseer, Alakija et al^[19]^; Alsharif and Al-Khaldi^[20]^ in Aseer and in Al-Madinah Al-Munawarah Al-Mosilhi and Kurashi^[18]^ reported that the among the most highly preferred means for fulfilling CME needs of PHC professionals were regular lectures (62.5%).

The present study indicated that hospital consultants were mostly preferred speakers by participants, followed by PHC physicians and university staff, while technical supervisors were the least preferred. Moreover, internet was the main source of information, followed by textbooks and their colleagues at PHC, while the least source for information was medical journals. Alsharif and Al-Khaldi^[20]^ reported that the most popular methods practiced by primary care physicians for CME were reading medical journals (79.8%) and medical textbooks (53.8%), while only few of them use the internet and email for CME purposes. They also reported that hospital consultants were the most preferred speakers in CME programs. Alakija et al^[20]^ in Aseer, reported that more than 2 thirds of PHC physicians prefer receiving CME from hospital consultants.

This study showed that 31.5% of participants had no or low improvement in their knowledge, 38.6% underwent intermediate improvement and 30% had high improvement. Regarding improved skills, 43.4% had no or low improvement, 45.3% had intermediate improvement and 11.2% had high improvement. These findings indicate the beneficial impact of CME on primary care professionals’ knowledge and skills and the need to improve CME programs to maximize expected benefits.

Findings of the present study showed that the main barriers against attending CME courses were the lack of time and the high workload. Similarly, it was reported that in Aseer, reported that the main barriers against attending CME by primary care physicians were work overload (42.5%) and lack of time (26.3%).^[20]^

Finding of the present study showed that CME attendance differed significantly according to participants’ position, with physicians being the most frequently attending and pharmacists being the least frequently attending. Moreover, participants’ knowledge improvement due to CME attendance differed significantly according to their position, with highest improvement among lab technicians. Participants’ skills improvement was highest among those aged > 40 years, among males, non-Saudis, those with Doctorate degrees, physicians, and those with more than 10 years’ experience. Personal characteristics were determinant factors regarding attending CME and its impact on knowledge and skills of health care providers. These differences indicate the need to encourage those who attend less CME activities than others and those whose CME activities yield less gain in knowledge or skills.

Several studies reported different findings regarding attending CME and its outcome. Alsharifin Aseer, reported that significantly more male physicians attended training courses than female physicians. Moreover, it was reported that physicians who used online CME programs were more likely to be younger and female in comparison to their older and male counterparts in their study sample.^[21]^ However, Lowe et al^[22]^ stated that CME activities are not limited to specific audience characteristics, by gender, age, or other similar variables.

## 10. Implications and contribution of the current study

This study analyzes the preferences, information sources, skill improvements, and attendance barriers among primary healthcare professionals in Abha City, Saudi Arabia. It identifies factors to enhance CME programs, provides insights for policymakers, and offers actionable recommendations for improving the continuing education framework within Saudi Arabian healthcare systems. The study identifies speaker preferences and information sources for tailored CME programs, enhancing their effectiveness. It also examines attendance barriers, providing insights for policymakers and healthcare administrators to improve CME accessibility in Saudi Arabia.

## 11. Limitations of the study

The study’s limitations suggest that response bias, where participants have different perspectives on CME, could have skewed the results, potentially resulting in an overrepresentation of healthcare professionals with stronger opinions.

Second, the study’s representativeness, focusing on primary healthcare professionals in Abha City, raises concerns about its potential inaccuracies in broader healthcare contexts, suggesting caution in its application to a broader population. Lastly, the study’s findings may not be universally applicable due to cultural differences in healthcare systems and preferences. Future research should explore CME needs and barriers in diverse settings to improve its applicability.

## 12. Conclusions

Approximately 2-thirds of PHC professionals attended CME courses, with attendance largely influenced by course topics, convenience of timing, and personal interests. Most participants found CME courses beneficial, primarily for enhancing their knowledge and skills. Lectures emerged as the preferred educational method, followed by videos and PowerPoint presentations, with hospital consultants being the most favored speakers. The primary sources of information for these professionals were the internet, textbooks, and colleagues. However, significant barriers to attendance were identified, particularly time constraints and high workloads. To address these issues, healthcare organizations should offer flexible scheduling options for CME courses and consider online or hybrid formats to accommodate busy professionals. Furthermore, focused outreach to certain groups, such as physicians and senior healthcare providers, may increase involvement and skill development, particularly among those with Doctorates and substantial experience. These measures can serve to maximize the effectiveness of CME and, eventually, improve the quality of care provided by primary healthcare workers.

## Acknowledgments

We would like to acknowledge the study participants who participated in this study.

## Author contributions

**Conceptualization:** Hassan Mohammed Hassan Alshehri.

**Formal analysis:** Hassan Mohammed Hassan Alshehri.

**Methodology:** Salem Mohammed Hassan Alshehri, Saleh Ahmed Alshaikhi.

**Resources:** Ali Mohammed Hassan Alshehri.

**Software:** Amer Mohammed Hassan Alshehri.

**Supervision:** Yousef H. Al Zahib, Hassan Mohammed Algarni.

**Visualization:** Ayedh A.A. Alahmari.

**Writing – review & editing:** Fahad Saeed Nasser Alasmari.

## Supplementary Material


