# Supplementary material for: Primary health care professionals’ opinions regarding continuing medical education: A cross sectional study
Source: Medicine (Baltimore). 2024 Dec 13;103(50):e40865. doi: 10.1097/MD.0000000000040865 (PMC11651445; doi:10.1097/MD.0000000000040865)
Supplement: Supplementary file 1 [file medi-103-e40865-s001.docx]

**Supplementary file (Study questionnaire)**

**A- Personal characteristics:**

- Age: Years
- Gender: 1- Male 2- Female
- Nationality: 1- Saudi 2- Non-Saudi
- Marital status: 1- Single 2- Married
- Position: 1- Physician 2- Pharmacist 3- Dentist 4- Lab tech. 5- Nurse
- Qualification: 1- Bachelor 2- MSc 3- Doctorate/ Fellowship 4- Other
- Years of experience in PHC: Years

**B- Continuing education**:

Did you attend continuing educational activity during last year? 1- Yes 2- No

- If yes, How many? ………………………..

- Factors that are important to you when selecting a continuing educational activity:

- Convenience of time
- Convenience of place
- Cost
- Personal interest
- Topic
- Presenter, who addresses the educational material
- Others (to be mentioned): ……………………..

Do you think that attending continuing education courses is important for you?

1- Yes 2- No

- If your answer is yes, WHY?
- Improves knowledge
- Improves skills
- It is obligatory to obtain the required CME hours
- Other, specify………………………………………………..
- If your answer is No, WHY?
- Waste of time
- No actual benefit
- Waste of money
- Other, specify………………………………………………..

Educational delivery methods you prefer most:

- Self-learning
- Conferences
- Lectures
- Powerpoint lectures
- Videos
- Case presentation
- Printed materials (e.g., books, journals)
- Multimedia educational materials (e.g., CD, DVD)
- Internet-based educational activities
- Open discussions
- Others (to be mentioned): ……………………..

Preferred speakers:

- Hospital consultants
- University staff
- Technical supervisors
- PHC physicians
- Others (to be mentioned): ……………………..

What are your main resources for continuing education?

- Textbooks
- Journals
- Internet
- Colleagues at PHC
- Others (to be mentioned): ……………………..

What are the barriers that you may face when selecting a continuing educational activity?

- Lack of time
- Family and social obligations
- Lack of money
- High workload
- Others (to be mentioned): ……………………..

How would you grade the effect of attending CME activities during the last year on your professional knowledge?

- None/low
- Intermediate
- High

How would you grade the effect of attending CME activities during the last year on your professional skills?

- None/low
- Intermediate
- High
